# Improved Odor Identification Ability and Increased Regional Gray Matter Volume After Olfactory Training in Patients With Idiopathic Olfactory Loss

**DOI:** 10.1177/20416695211005811

**Published:** 2021-04-22

**Authors:** Pengfei Han, Martina Musch, Nasreddin Abolmaali, Thomas Hummel

**Affiliations:** Interdisciplinary Center Smell and Taste, Department of Otorhinolaryngology, Medical Faculty Carl Gustav Carus, TU Dresden, Dresden, Germany Faculty of Psychology, 26463 Southwest University, Chongqing, China; Interdisciplinary Center Smell and Taste, Department of Otorhinolaryngology, Medical Faculty Carl Gustav Carus, 9169 TU Dresden, Dresden, Germany; Municipal Hospital Friedrichstadt, 9169 TU Dresden, Germany; Interdisciplinary Center Smell and Taste, Department of Otorhinolaryngology, Medical Faculty Carl Gustav Carus, 9169 TU Dresden, Dresden, Germany

**Keywords:** idiopathic olfactory loss, olfactory training, odor identification, gray matter volume, severity of olfactory loss

## Abstract

Idiopathic olfactory loss (IOL) is thought as an early marker for neurodegenerative disease. This study investigated the effect of olfactory training (OT) on regional gray matter volume (GMV) among patients with IOL. A total of 24 patients (mean age 64.6 years, 11 male) with IOL and 30 control participants with normal olfaction (mean age 62.6 years, 13 males) were included in the study. Voxel-based morphometry was performed to compare the GMV between patient and control groups. Only the patients received OT (averaged duration 7 months), and a longitudinal approach was used to examine the GMV change from pre- to post-OT. Moreover, the effect of OT on GMV change was explored for patients with different severity of olfactory loss (anosmia vs. hyposmia). Olfactory performance was measured alongside using the “Sniffin’ Sticks.” Patients had improved odor identification and larger GMV in the bilateral cerebellum, bilateral thalamus, left precentral gyrus, right gyrus rectus, and medial orbitofrontal cortex after OT. However, no correlation was found between changes of odor identification and increased regional GMV. Besides, patients with anosmia, compared with patient with hyposmia, demonstrated increased GMV in the left precuneus, left superior frontal medial cortex, and left midcingulate cortex after OT. The study showed improved odor identification ability among patients with IOL after OT, which is unlikely related to spontaneous recovery. In this specific patient group, the GMV alterations may be associated with factors not directly predicted by the currently performed measurements, but possibly higher order olfactory-related functional changes.

Olfactory dysfunction is an increasingly recognized form of sensory impairment. It is estimated that one fifth of the general population has a decreased sense of smell including approximately 5% with little or no olfactory function (Hummel et al., 2016). Apart from aging, major causes for olfactory dysfunction include upper respiratory tract infection, sinonasal disease, trauma, idiopathic causes, and congenital anosmia (Temmel et al., 2002). Patients are diagnosed as idiopathic olfactory loss (IOL) when no cause has been found with detailed clinical investigations (including questionnaires, psychophysical olfactory testing, and olfactory pathways morphology assessment; Rombaux et al., 2010). Because it has an unknown etiology, the IOL is also regarded as an early sign for idiopathic Parkinson’s disease (Haehner et al., 2007, 2019) or Alzheimer’s disease (Koss et al., 1988).

Acquired olfactory loss is related to alterations of the central olfactory system, including but not limited to attenuated brain responses to odor stimuli, reduction of gray matter volume (GMV) and white matter volume, or decreased olfactory bulb (OB) size (Han et al., 2019). Cross-sectional studies have shown that patients with IOL have smaller OB (Rombaux et al., 2010) and brain regional GMV (Yao et al., 2014) when compared with control participants with normal olfaction.

Among available treatments, the olfactory training (OT) has been suggested to be an effective way to improve olfactory performance (Sorokowska et al., 2017). The human peripheral and central olfactory system are highly plastic (Reichert & Schopf, 2017). For patients with olfactory dysfunctions, OT leads to improved psychophysical olfactory assessment scores (Hummel et al., 2009), increased OB volume, enhanced brain responses to odor stimuli (Pellegrino et al., 2019), or altered brain connectivity (Kollndorfer et al., 2015). Patients with olfactory loss due to upper respiratory tract infection exhibit increased GMV after 3 months of OT. The observed brain regions include parahippocampal gyrus, thalamus, and cerebellum (Gellrich et al., 2018). Besides, increased volume of the OB had been shown after OT among people with normal olfactory function (Negoias et al., 2017). Further, 6 weeks of intensive (20 minutes per day) OT among healthy participants showed increased cortical thickness in brain areas involved in olfaction, including the right inferior frontal gyrus and right entorhinal cortex, and bilateral fusiform gyri (Al Ain et al., 2019). However, most of the previous studies had included heterogeneous patients with mixed causes for their olfactory loss. The effect of OT on olfactory function improvement and brain structural modulation among patients with IOL is unclear.

The current study was aimed to investigate the effect of OT on olfactory performances and brain regional GMV of patients with IOL. Moreover, the study also explored whether the effect of OT will differ as a function of the olfactory loss severity (e.g., between patients with hyposmia and patients with anosmia).

## Material and Method

### Participants

Twenty-six patients with IOL and 32 control participants were initially included in the study. The following participants were excluded: one patient with normal olfaction (Sniffin’ Sticks TDI score = 30.75); one patient with blurred raw magnetic resonance imaging (MRI) image; one control participant with abnormal olfactory function (TDI = 21.5); and one control participant with corrupted MRI images. Therefore, 24 patients with IOL (mean age = 64.6 years, *SD* = 10.2, range from 44 to 79 years; 11 males) and 30 control participants (mean age = 62.6 years, *SD* = 15.2, range from 22 to 82 years; 13 males) were included in the final study. Olfactory loss was due to an unidentifiable cause after an extensive clinical examination, including detailed and structured history, nasal endoscopy, documentation of missing responsiveness to topical or systemic steroids (indicating absence of inflammatory causes of olfactory loss), and detailed evaluation of smell and taste function. The patients had an average olfactory loss duration of 4.9 years (range from 1 to 17 years). Olfactory function was tested using the “Sniffin’ Sticks” test battery (Hummel et al., 1997; Kobal et al., 1996). The combined odor threshold, odor discrimination, and odor identification score (TDI score) was used to separate normosmia (TDI > 30.5) from hyposmia (16.5 < TDI < 30.5) or functional anosmia (TDI < 16.5; further termed “anosmia”). Among the patients, 12 had idiopathic hyposmia (mean age = 63.3 years, *SD* = 11.5, 6 males) and 12 had idiopathic anosmia (mean age = 65.8 years, *SD* = 9.0, 5 males). One patient was diagnosed with phantosmia, one patient with parosmia, and one with both parosmia and phantosmia. The control and the patient groups matched in terms of age, *t*(_52_) = 0.54, *p* = .59, and sex distribution, χ^2^ = 0.034, *p* = .54. The hyposmia and anosmia patient subgroups matched in terms of age, *t*(_22_) = 0.59, *p* = .50, and sex distribution, χ^2^ = 0.17, *p* = .68. This study was approved by the Ethics Committee at the Medical Faculty of the TU Dresden. All participants gave informed, written consent.

### OT Protocol

The patients completed a commonly used OT protocol (Hummel et al., 2009), that is, bi-daily smelling of four different odorants: phenyl ethyl alcohol (rose), eucalyptol (eucalyptus), citronellal (lemon), and eugenol (cloves). Patients were instructed to smell each of the four odors individually, twice per day (once in the morning, once at night), by holding each jar under their nostrils, and sniffing for a period of 20 to 30 seconds each. Patients were equipped with the odorants, which were contained in small glass jars with a screw-on lid. The average duration of OT was 7 months, with a range of 6 to 9 months. The control group did not receive OT.

### Structure Image Acquisition

The MRI scans were performed on a 3T GE scanner (General Electrics Healthcare, Milwaukee, WI, USA) with a 12-channel phase-array head coil. Structural T1-weighted images of the participants were acquired using a 3D magnetization prepared gradient rapid acquisition gradient echo (MPRAGE) sequence, with the following parameters: TR 2530 ms; TE 2.34 ms; TI 1100 ms; field of view 256 mm; voxel size 1 × 1 × 1 mm; flip angle 7°, and 192 contiguous slices of 1 mm thickness. Images were acquired in the axial plane oriented parallel to the planum sphenoidale to minimize artifacts.

### Voxel-Based Morphometry

Voxel-based morphometry of T1-weighted images was performed using the CAT12 software (http://dbm.neuro.uni-jena.de/vbm/) implemented in SPM12 (Wellcome Centre of Imaging Neuroscience, Institute of Neurology, UCL, London, UK; http://www.fil.ion.ucl.ac.uk/spm) and MATLAB (version 2013a, The MathWorks, Natick, MA, USA). T1 images were first segmented into gray matter (GM), white matter (WM), and cerebrospinal fluid. A two-step segmentation approach was applied: First, the normal segmentation in CAT12 was used for T1 images from the control group and the patient group before OT; second, the T1 images from the patient group (pre- and post-OT) was segmented using the method for longitudinal data in CAT12.

After segmentation, the GM images were spatially normalized to a template in Montreal Neurological Institute (MNI) space using the high-dimensional Diffeomorphic Anatomical Registration Through Exponentiated Lie Algebra (DARTEL; Ashburner & Friston, 2000). The quality control in CAT12 was applied to ensure high quality of the segmentation of GM from WM. Mean correlation measures the homogeneity of the unsmoothed GM segments served as an indicator for data quality after preprocessing. This tool visualizes the correlation between the volumes using a boxplot and correlation matrices. Outliers (deviated greater than two standard deviations from the global mean) were excluded from the sample before smoothing and statistical analyses. For both groups, no data were indicated as having a mean correlation more than two standard deviations. Finally, the normalized GM images were smoothed with a Gaussian kernel (full width at half maximum 8 mm). In addition, an absolute threshold masking value of 0.2 was applied to avoid possible edge effects between different tissue types (Ashburner & Friston, 2000). The volumes of GM, WM, and cerebrospinal fluid of each participant were summed up to the total intracranial volume (TIV).

GMV between the control and patient groups was examined using the two-sample *t*-test model in SPM12, including TIV as covariate. To compare the GMV of the patient groups between the pre-OT and post-OT sessions, a flexible factorial design was specified, including the subject factor (24 levels: 24 subjects; independence and equal variance assumed between-subjects) and the training factor (2 levels: pre-OT and post-OT; dependence and equal variance assumed between levels). Besides, to investigate the varied effect of OT on GMV change in patients with different severity of olfactory loss, a flexible factorial design was specified, including the subject factor (24 levels: 24 subjects; independence and equal variance assumed between-subjects), the severity factor (2 levels: hyposmia, anosmia; independence and unequal variance assumed between groups), and the training factor (2 levels: pre-OT, post-OT; dependence and equal variance assumed between levels). For longitudinal models, age, TIV, and the training time (in month) were included as covariates. As covariate inclusion during voxel-based morphometry analyses has strong impact on the results (Hyatt et al., 2020), we also performed the same analyses without the aforementioned covariate (age, TIV, or training time). As there was little change for the outcome, the results without covariates were not reported.

Statistical analysis was conducted on a whole-brain level. A cluster-extent-based threshold of *p* < .001 (uncorrected) was considered as statistically significant (Woo et al., 2014) and was used in combination with a nonstationary threshold to balance the risks of Type-I and Type-II errors (Lieberman & Cunningham, 2009). To control for multiple statistical testing within the entire brain, we maintained a cluster-level false-positive detection rate at *p* < .05 using an initial voxel-level threshold of *p* < .001 with a cluster extent (k) empirically determined by Monte Carlo simulations (*n* = 1,000 iterations), by means of AlphaSim procedure (Forman et al., 1995). This was done using the REST toolbox (http://www.restfmri.net/forum/REST_V1.7; Song et al., 2011). The following cluster size was determined to achieve a cluster-level family-wise error corrected *p* < .05: k = 31 voxels for comparison between control and patient groups; k = 44 voxels for comparison between pre-OT and post-OT in patient group; and k = 36 voxel for testing the effect of OT on patients with differed disease severity. Significant brain regions were labelled and reported with the AAL3 toolbox (Tzourio-Mazoyer et al., 2002).

### Statistical Analysis for Behavioral Data

The olfactory function data as measured by the Sniffin’ Sticks test were analyzed using SPSS (Statistical Packages for Social Sciences, version 25, SPSS Inc., Chicago, IL, USA). TDI scores for patients and controls in the pre-OT session were compared using one-way analysis of variance (ANOVA). The odor threshold, odor discrimination, and odor identification scores between the pre- and post-OT sessions in the patient group were analyzed using three by two repeated-measure ANOVA. A Bonferroni correction was used for post hoc tests to control for Type-1 errors due to multiple comparisons. The alpha level was set at *p* = .05 for all statistical tests.

## Results

### Effect of OT on Olfactory Performances Among Patients

**Figure 1.**
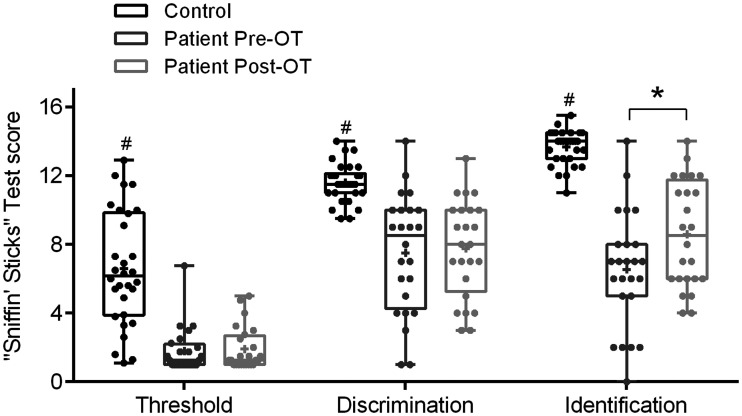
The “Sniffin’ Sticks” test scores of the control group (*N* = 30) and the patient group (*N* = 24) in the pre- and post-olfactory training sessions. In each whisker plot, the central line denotes the median; the plus (+) denotes the mean. The upper and lower edge of the box represents the minimum to maximum value. # indicates significant higher test scores (*p* < .001) for the control compared with patient group; *indicates significant improved odor identification scores (*p* < .05) for the patient group after training. OT = olfactory training; Pre-OT = pre-olfactory training; Post-OT = post-olfactory training.

Before OT, controls had significantly better olfactory functioning than the patients in terms of odor thresholds, *F*(1, 52) = 44.91, *p* < .001; odor discrimination, *F*(1, 52) = 37.29, *p* < .001; and odor identification, *F*(1, 52) = 121.74, *p* < .001 (Figure 1).

For patient group, the repeated-measure ANOVA showed a significant interaction between OT and type of olfactory function, *F*(2, 46) = 4.46, *p* = .02. The post hoc analysis showed that the odor identification score was significantly improved after OT among the patient group (pre-OT mean = 6.5, *SD* = 3.4; post-OT mean = 8.6, *SD* = 3.1; *F*(1, 23) = 12.30, *p* = .002), but not for odor threshold or odor discrimination scores (Figure 1).

### Regional GMV in Control and Patient Group Before OT

Compared with the control group, patients with IOL demonstrated larger GMV in the lateral orbitofrontal cortex (OFC; MNI peak coordinates xyz = 44 54 –21; peak T = 4.15; cluster size = 40 voxels). However, no significantly larger GMV in control participants when compared with patient participants was observed.

### Effect of OT on Regional GMV

**Figure 2.**
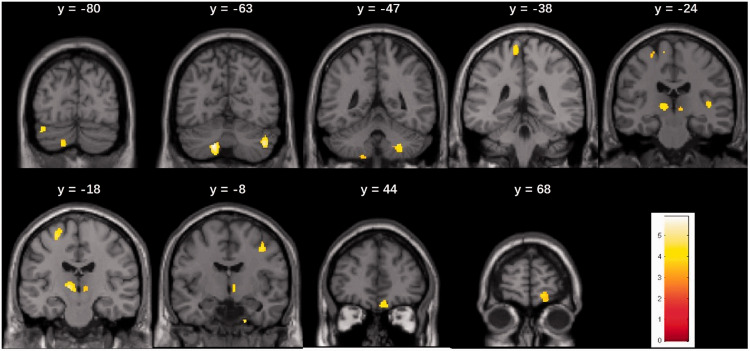
Brain areas in patients with idiopathic olfactory dysfunction showing increased gray matter volume after olfactory training. Results are significant at uncorrected *p* < .001 with cluster-extent threshold of k > 44 contiguous voxels.

After OT, increased GMV was found in the bilateral cerebellum, the bilateral thalamus, the left precentral gyrus and thalamus, also in the right superior frontal cortex, right supplementary motor area, right medial OFC, and the right gyrus rectus (Table 1, Figure 2). The opposite contrast revealed significantly larger GMV in the pre-OT session in the cerebellum, frontal cortex, angular gyrus, and the inferior operculum, all identified in the right hemisphere (Table 1).

**Table 1.** Comparison of Regional Gray Matter Volume in Patients With Idiopathic Olfactory Dysfunction Before and After Olfactory Training.

	k	T	xyz			Region
Pre-OT > Post-OT	640	6.68	20	–62	–29	Cerebellum R
	196	4.80	59	2	42	Middle frontal cortex R
	190	4.80	–3	45	48	Superior medial frontal cortex R
	96	4.60	47	42	29	Middle frontal cortex R
	116	4.40	60	–36	50	Angular gyrus R
	84	4.30	41	20	57	Middle frontal cortex R
	233	4.23	14	42	54	Superior medial frontal cortex R
	59	3.78	62	15	15	Frontal inferior operculum R
Post-OT > Pre-OT	629	5.85	–17	–63	–42	Cerebellum L
	277	5.16	44	–65	–41	Cerebellum R
	321	4.93	–11	–18	2	Thalamus L
	160	4.66	–24	–18	63	Precentral gyrus L
	147	4.55	41	–8	45	Superior frontal cortex R
	125	4.41	–41	–80	–24	Cerebellum L
	128	4.31	27	–47	–45	Cerebellum R
	240	4.28	17	68	–11	Medial orbitofrontal cortex R
	255	4.26	8	44	–21	Gyrus recuts R
	80	4.15	18	–6	–41	Fusiform gyrus R
	170	4.14	–9	–38	69	Paracentral lobule L
	67	4.04	42	–24	5	Superior temporal cortex R
	244	3.93	8	–8	–3	Thalamus R
	46	3.60	–17	–48	–57	Cerebellum L

*Note*. All reported results were significant at uncorrected *p* < .001 and cluster size of k > 44 contiguous voxels across the whole brain. Pre-OT = pre-olfactory training; Post-OT = post-olfactory training.

### Effect of OT for Patients With Different Severity of Olfactory Loss

A significant Severity × Training interaction effect was found, showing that patients with anosmia compared with patient with hyposmia had increased GMV in the left precuneus, left superior frontal medial cortex, and the left midcingulate cortex after OT (Table 2). However, no significant effect of Training × Severity was observed for olfactory functions measured by the “Sniffin’ Sticks” test.

**Table 2.** Significant Effects of Olfactory Training on Gray Matter Volume Change in Patients With Varied Severity of Olfactory Loss.

	k	T	xyz			Region
Anosmia (Post-OT > Pre-OT)—Hyposmia (Post-OT > Pre-OT)	311	5.45	–11	–56	30	Precuneus L
	105	5.03	–12	66	12	Superior medial frontal cortex L
	38	4.35	–12	–33	41	Middle cingulate cortex L
Hyposmia (Post-OT > Pre-OT)—Anosmia (Post-OT > Pre-OT)	–	–	–	–	–	–

*Note*. All reported results were significant at uncorrected *p* < .001 and cluster size of k > 36 contiguous voxels across the whole brain. Pre-OT = pre-olfactory training; Post-OT = post-olfactory training.

### Correlations

There was no significant correlation (*p* > .1 for all Pearson’s correlation tests) between the increased GMV in the identified clusters and the change of odor identification scores among patients.

## Discussion

In the current study, we investigated the effect of OT on regional GMV changes in a group of patients with IOL. After a minimum 6 months of OT, the patients showed increased GMV of the cerebellum, thalamus, precentral gyrus, paracentral lobule, medial OFC, and gyrus rectus. Some of the observed brain regions (e.g., the thalamus and cerebellum) are comparable to previous findings among olfactory-loss patients with mixed causes who received OT (Gellrich et al., 2018).

The patients showed significantly improved odor identification ability after OT, which is congruent with the literature on the effectiveness of OT for patients with olfactory dysfunction: OT mainly improves performance in higher order tasks such as odor identification or discrimination rather than odor detection thresholds (Sorokowska et al., 2017). Because of the long-standing olfactory loss of the patients, it is unlikely that the improvement of olfactory function was due to spontaneous recovery. However, in the present study, a lack of correlation between improved odor identification performances and changes of regional GMV indicated an unidentified mechanism. Similarly, a recent study on participants with normal olfaction reported no correlations between changed olfactory test scores and alteration of brain structure after extensive OT. Instead, the study reported a trended correlation between odor memory and structural brain changes (Al Ain et al., 2019). Thus, one possible interpretation of the present and previous relevant findings is that the brain alterations after OT are mediated by higher order cognitive factors. Besides, increased GMV of the precentral gyrus, which is the primary motor area, was observed in the current study and the study by Al Ain (Al Ain et al., 2019). One plausible reason for this finding and other observed results in the current study (e.g., the increased GMV of the paracentral lobule and the cerebellum) is the effect from olfactomotor performances during OT (Mainland et al., 2005; Sobel et al., 1998). However, a previous OT study using minimal odor concentrations did not lead to an increase in olfactory function in comparison to the use of clearly perceptible odors with higher concentrations, arguing against the idea that sniffing alone improves the sense of smell (Damm et al., 2014).

Several other brain regions that showed increased GMV after OT are implicated in higher order olfactory-related processes. For example, the thalamus is associated with olfactory attention (Zobel et al., 2010). The fusiform gyrus is involved in odor memory (Cerf-Ducastel & Murphy, 2006; Kjelvik et al., 2012). The gyrus rectus is part of the olfactory system (Fjaeldstad et al., 2017), especially with regards to olfactory-related higher order cognitive processes. Perfumers who have long-term professional odor training demonstrated increased GMV of the gyrus rectus, and their GMV of the medial OFC was positively correlated with the years of experience among professional perfumers (Delon-Martin et al., 2013). Taken together, it is speculated that the increase of regional GMV after OT among patients with IOL is related more to a top-down rather than bottom-up modulation (Pellegrino et al., 2019). As indicated by the current study results, these top-down effects have limited direct association with typical olfactory functions (e.g., sensitivity, discrimination or identification).

Moreover, compared with patients with idiopathic hyposmia, patients with idiopathic anosmia showed significantly increased GMV in the precuneus, the superior frontal medial cortex, and the midcingulate cortex after OT, all in the left hemisphere. Although not characterized as the main olfactory areas, these regions were suggested in relation to olfactory memory (Arshamian et al., 2013; Savic & Berglund, 2004). This finding suggests that the level of OT-related plasticity among patients with IOL may be associated with baseline olfactory functions.

Several study limitations need to be noted. First, the study lacks an OT control group; therefore, it is difficult to determine the causes for the post-OT GMV changes (including reduction or increase) observed in the patient group. A preferable design is to have a separate group of patients being scanned twice at the same time points but without having conducted OT. A further limitation is related to the compliance to OT. For example, the long duration of training increases the chances of losing participants with the attrition rate as high as 45% in some 6-month studies (Lamira et al., 2019), although this was not the case in the present study. Compliance is important as one recent study of trigeminal training showed that the correlation between training time and improvement of trigeminal sensitivity became insignificant after controlled for the compliance (Oleszkiewicz et al., 2018). Although the actual compliance was assumed to be highly based on the personal interviews with the patients following OT, the inclusion of training diaries or a modified training device (Saatci et al., 2020) should be considered in future research.

In conclusion, the current study showed that in a group of patients with IOL, OT is associated with improved odor identification ability and regional GMV in several brain areas. However, in this specific group of patients, the alteration of GMV may be associated with factors not predicted by the currently performed measurements but likely to be mediated by higher order brain functional changes related to odor-related memory, or attention.
